# Carboxymethyl Cellulose/Copper Oxide–Titanium Oxide Based Nanocatalyst Beads for the Reduction of Organic and Inorganic Pollutants

**DOI:** 10.3390/polym15061502

**Published:** 2023-03-17

**Authors:** Esraa M. Bakhsh, Sher Bahadar Khan, Nujud Maslamani, Ekram Y. Danish, Kalsoom Akhtar, Abdullah M. Asiri

**Affiliations:** 1 Chemistry Department, Faculty of Science, King Abdulaziz University, P.O. Box 80203, Jeddah 21589, Saudi Arabia; 2 Chemistry Department, College of Science, Imam Abdulrahman Bin Faisal University, P.O. Box 76971, Dammam 31441, Saudi Arabia; 3 Center of Excellence for Advanced Materials Research, King Abdulaziz University, P.O. Box 80203, Jeddah 21589, Saudi Arabia

**Keywords:** catalytic reduction, carboxymethyl cellulose, nanocatalyst, catalytic reduction, organic and inorganic pollutants

## Abstract

In this work, we have developed novel beads based on carboxymethyl cellulose (CMC) encapsulated copper oxide-titanium oxide (CuO-TiO_2_) nanocomposite (CMC/CuO-TiO_2_) via Al^+3^ cross-linking agent. The developed CMC/CuO-TiO_2_ beads were applied as a promising catalyst for the catalytic reduction of organic and inorganic contaminants; nitrophenols (NP), methyl orange (MO), eosin yellow (EY) and potassium hexacyanoferrate (K_3_[Fe(CN)_6_]) in the presence of reducing agent (NaBH_4_). CMC/CuO-TiO_2_ nanocatalyst beads exhibited excellent catalytic activity in the reduction of all selected pollutants (4-NP, 2-NP, 2,6-DNP, MO, EY and K_3_[Fe(CN)_6_]). Further, the catalytic activity of beads was optimized toward 4-nitrophenol with varying its concentrations and testing different concentrations of NaBH_4_. Beads stability, reusability, and loss in catalytic activity were investigated using the recyclability method, in which the CMC/CuO-TiO_2_ nanocomposite beads were tested several times for the reduction of 4-NP. As a result, the designed CMC/CuO-TiO_2_ nanocomposite beads are strong, stable, and their catalytic activity has been proven.

## 1. Introduction

Large quantities of organic and inorganic contaminates released to the ecosystem through wastewater steam due to different human activities (e.g., industrial waste, textile, agrochemical and pharmaceutical). These organic and inorganic pollutants, such as dyes, nitrophenols, and potassium hexacyanoferrate are naturally unmanageable, highly toxic, hazardous in nature, carcinogenic, mutagenic, and limited biodegradable. They are also seen as being harmful and dangerous to living things [1,2,3,4,5].

As a result, the techniques for removing these types of pollutants have significant drawbacks, such as low elimination rates, high cost and complexity, and slow removal efficiency, which limit their uses. Many efforts have been devoted to remove toxic contaminants from water bodies, but some of the established methods are not useful enough to complete the removal of toxic pollutants, besides long time consumption and their high cost. The demand for pollution management around the world is to develop new cost-effective, environmentally friendly, simple, and new manageable processes to remove the toxic contaminants or convert into the toxins into useful compounds. The rising advancement of usage of green chemistry in pollution management is due to the fact that environmentally friendly processes have a long history of being both environmentally and economically beneficial [6,7,8]. Recently, huge efforts have been made to develop methods based on catalytic reduction/degradation/transformation of these toxic contaminates to less toxic form and useful compounds which is a good way to deal with wastewater contaminates. Catalytic reduction is one strategy that requires less time to remove the toxic pollutants, which is easy, fast and involves a very low quantity of solvent compared to other techniques. According to literature, various catalysts have been prepared from an efficient, stable, and selective material to be an efficient for the removal toxic organic and inorganic contaminates from wastewater. 

Copper oxide nanocatalyst can achieve good selectivity without any extra additives for reducing nitrophenols and dyes. Due to its unique characteristics, such as a high surface-to-volume ratio and higher activity compared to those of the bulk materials [9,10,11]. Titanium dioxide (TiO_2_) is also frequently used as a nanocatalyst or substrate for immobilizing metal nanomaterial because of its favorable qualities of non-toxicity, stability, and hydroxyl-rich surface [12]. In particular, copper oxide (p-type) and titanium oxide (n-type) are an important class of semiconductor for efficient dye degradation processes and the reduction of nitrophenols. Thus, a nanocatalyst based on the combination of TiO_2_ and CuO have been developed for the reduction of nitrophenol and dyes due to their great advantages of adjustable oxidation states, low cost, as well as thermal and chemical stability [13,14,15,16,17,18]. Unfortunately, the powder nanocatalyst of metal oxides encountered issues like aggregation, difficulties in separation and reuse of these materials [14,19,20,21,22,23].

Polymer-based metal oxide nanocomposite holds great promise as a viable approach to overcome the aforementioned challenges of metal oxide nanoparticles. According to recent studies, different polymer-based nanocomposites have been fabricated by various ways, either by coating or hosting the fine nanomaterial onto the polymer matrix of larger size. Polymeric host materials are an attractive route to control the pore space and surface of the nanomaterials as well as their excellent properties, which could improve the mechanical strength for long-term use. Several polymers, including cellulose or carboxymethyl cellulose [13,24,25,26,27,28], chitosan, agarose, clay, alginate and so on [21,29,30,31,32,33,34,35,36], are used. Among them, CMC has been proven to be an appropriate polymer host for various metal oxide nanocomposites.

In the current study, an efficient nanocatalyst beads were developed based on incorporating the nanocomposite CuO-TiO_2_ onto the CMC as a host polymer by the help of cross-linking agent AlCl_3_. CMC/CuO-TiO_2_ beads were characterized by SEM, XRD and EDS. The synthesized CMC/CuO-TiO_2_ beads were evaluated as catalyst for the reduction of selected organic and inorganic pollutants.

## 2. Experimental

### 2.1. Chemicals and Reagents

Titanium (VI) oxide ((TiO_2_) of particle size < 100 nm and purity of >97% has been provided by Sigma Aldrich. Copper (II) nitrate (Cu(NO_3_)_2_), nitrophenols including 4-nitrophenol (4-NP), 2-nitrophenol (2-NP) and 2,6-dinitrophenol (2,6-DNP), organic dyes involving methyl orange (MO) and eosin yellow (EY), potassium hexacyanoferrate K_3_[Fe(CN)_6_], sodium borohydride (NaBH_4_), and carboxymethyl cellulose (CMC) were all purchased from Sigma-Aldrich. Distilled water was used in all experiments.

### 2.2. Synthesis of CuO-TiO_2_ Nanoparticles

CuO-TiO_2_ nanoparticles were prepared by dissolving cupric nitrate in distilled water (1:1 weight) and stirring thoroughly to completely dissolve the cupric nitrate. Then, the TiO_2_ was added to cupric nitrate solution (1:1 weight). After that, NaOH was added to elevate the pH of the liquid to 10. The resulting mixture was agitated overnight at 60 °C, then rinsed and dried multiple times. The precipitate was then calcined for 5 h at 500 °C.

### 2.3. Preparation of CMC/CuO-TiO_2_ Nanocatalyst Beads

**Figure 1:**
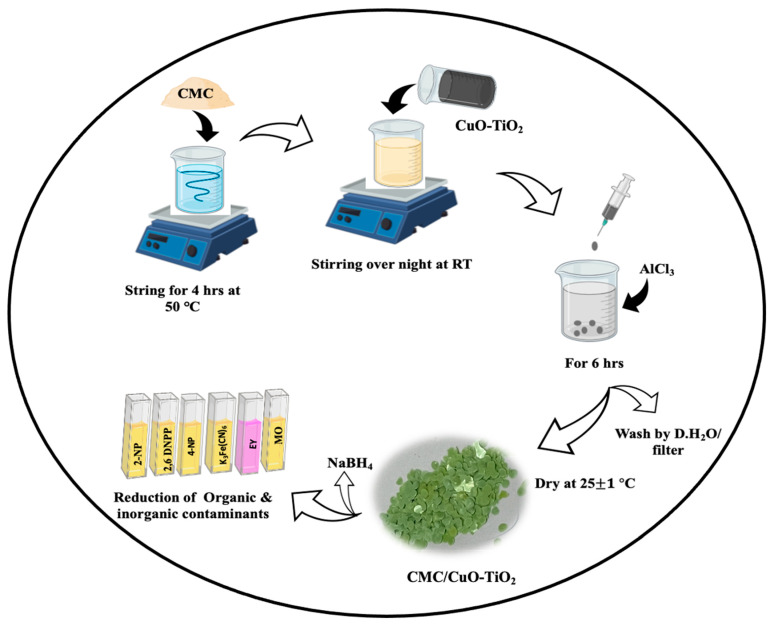
Schematic representation for the preparation of CMC/CuO-TiO_2_ nanocatalyst beads.

CuO-TiO_2_ nanoparticles were dispersed in CMC solution to make CMC/CuO-TiO_2_ nanocatalyst beads. Firstly, 0.1 g of CMC powder was dissolved in 50 mL of deionized water at 50 °C with stirring for 4 h. After CMC completely dissolved, 150 mg of CuO-TiO_2_ nanoparticles was added to the CMC solution and continuous stirring overnight at RT (25 ± 1), which resulted in a viscous suspension. The mixture was filled in syringe and loaded by dropwise into 0.2 M AlCl_3_, which used as a cross linker agent to form beads of CMC/CuO-TiO_2_. The CMC/CuO-TiO_2_ beads were kept in a crosslinking agent solution overnight for complete crosslinking. Afterward, the prepared beads were separated and washed several times with deionized water to eliminate the excess of unreacted Al^+3^ on the surface of the beads. Finally, the CMC/CuO-TiO_2_ beads were dried at a temperature of 25 °C on the pinch top, as shown in Figure 1.

### 2.4. Characterization

To evaluate the phase structure of the produced catalysts, X-ray diffraction (XRD) was used to confirm the morphologies and structures of CuO-TiO_2_ and CMC/CuO-TiO_2_. CuO-TiO_2_ and CMC/CuO-TiO_2_ were also analyzed using a scanning electron microscope (SEM) (JEOL, JSM-7600F, Akishima-shi, Japan) and were individually glued on the stub using carbon tape as a binder and then sputtered with platinum for 15 s. For elemental analysis, EDS was used, which is connected directly with the SEM. For UV–Vis spectra, a Thermo Scientific TM Evolution TM 350 UV–vis spectrophotometer was used to record the catalytic reduction studies.

### 2.5. Catalytic Reduction

The catalytic behavior of the developed nanocatalyst beads (CMC/CuO-TiO_2_) was tested with organic pollutants such as nitrophenol isomers [4-NP, 2-NP, and 2,6-DNP] and organic dyes (EY and MO) as well as inorganic compound K_3_[Fe(CN)_6_]. All these selected compounds were prepared in deionized water. In all catalytic reduction investigations, 2.5 mL of a pollutant solution was placed in the UV cuvette cell and passed across its UV-vis spectrum. After that, 0.5 mL of fresh reducing agent (0.1 M NaBH_4_) was added, followed by 5 mg of CMC/CuO-TiO_2_ beads, and the UV-vis absorption spectrum was continually recorded every 1.0 min. The percent % *reduction* of all compounds was calculated by utilizing Equation (1):

(1) $$\%Reduction=\frac{C_{0}-C_{t}}{C_{0}}\;*\;100$$

where *C*_0_ and *C_t_* are the initial and final concentrations of the studied compounds [6].

## 3. Result and Discussion

### 3.1. Characterization

#### 3.1.1. Scanning Electron Microscope (SEM)

**Figure 2:**
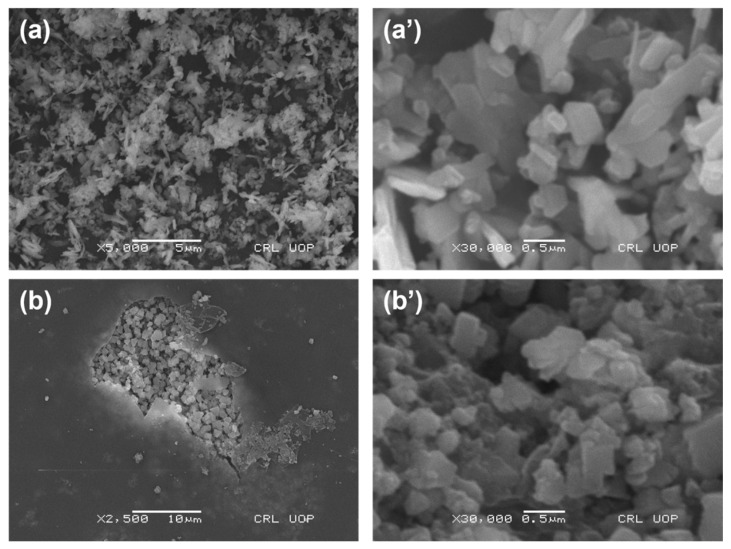
SEM images of (**a**,**a’**) CuO-TiO_2_ and (**b**,**b’**) CMC/CuO-TiO_2_.

The surface morphology of the prepared materials CuO-TiO_2_ and CMC/CuO-TiO_2_ was examined using SEM. Low-to-high-magnified SEM pictures for the prepared nanocomposites are represented on the left and right sides of Figure 2. Images of Figure 2a,a’ indicate the particles of CuO-TiO_2_ which look like aggregated nanosheets [37,38]. The pure beads of CMC show flat surfaces with less porousness, which was presented in our previous studies [14,31]. On the other hand, CMC/CuO-TiO_2_ was planted well on the CMC matrix, as observed in Figure 2b,b’. The surface of the CMC/CuO-TiO_2_ beads looks smooth due to the CMC while the aggregated particles appeared due to CuO-TiO_2_.

#### 3.1.2. X-ray Diffraction (XRD)

**Figure 3:**
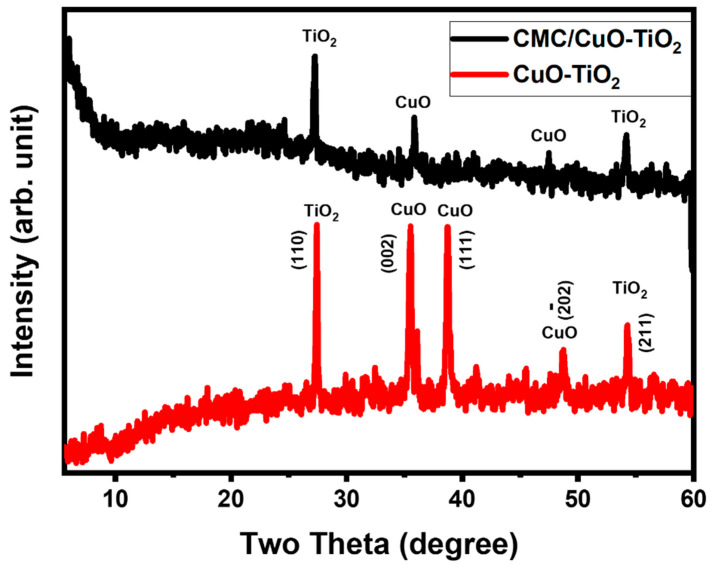
XRD pattern of CuO-TiO_2_ and CMC/CuO-TiO_2_.

The crystal structures and phase purities of CMC/CuO-TiO_2_ nanocomposite beads and CuO-TiO_2_ were tested by XRD analysis. The CuO-TiO_2_ nanocomposite pattern illustrated several diffraction peaks, which were indications for the CuO and TiO_2_ phases. As shown in Figure 3, the diffraction peaks at 2θ were equal to 27° and 55°, confirming the TiO_2_ in the rutile phase [39,40,41], whereas the diffraction bands at 2θ = 35.6°, 38.6°, and 48.8°, indicating the monoclinic structure of CuO [42]. As per our previous studies [1,38], the XRD pattern of CMC/CuO-TiO_2_ bead showed one additional small hump at 2θ = 23° which suggest the presence of amorphous phase of CMC present in the bead. The developed CMC/CuO-TiO_2_ nanocomposite beads has the same diffraction peaks, which indicating that CuO-TiO_2_ was planned very well in the CMC matrix as clearly seen from Figure 3.

#### 3.1.3. EDS Analysis

**Figure 4:**
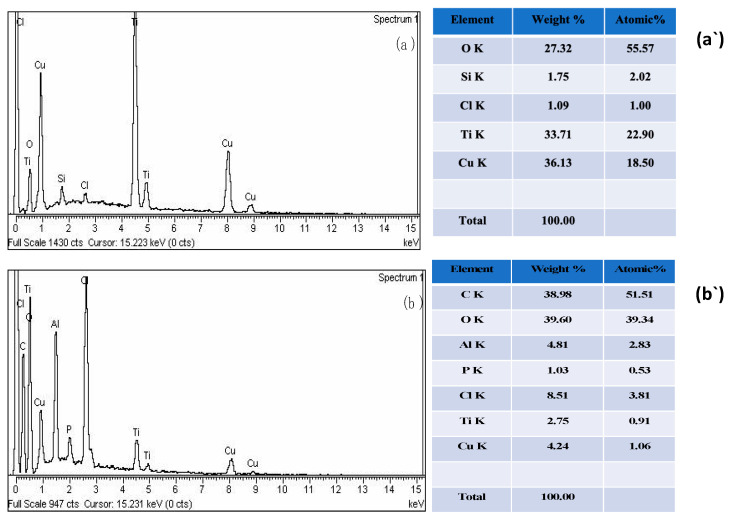
EDS spectrum of (**a**,**a’**) CuO-TiO_2_ and (**b**,**b’**) CMC/CuO-TiO_2_.

As shown in Figure 4a,a’, EDS was used to confirm the composition of CuO-TiO_2_ and CMC/CuO-TiO_2_ nanocatalysts. Copper (Cu), titanium (Ti), and oxygen (O) peaks were visible in the EDS spectra of the CuO-TiO_2_ nanoparticles. Cu peaks were found at 0.9, 8.0, and 9.0 keV, whereas Ti peaks were found at 0.5, 4.5, and 5.0 keV, and O peaks were at 0.48 keV. Referring to Cu, Ti, and O, the data indicated the production of CuO-TiO_2_ nanoparticles. Thus, the CuO-TiO_2_ nanoparticles were made up of Cu, Ti, and O, according to EDS. The oxygen content was 27.32 wt% while Cu and Ti were 36.13 wt% and 33.71 wt%, according to EDS data. At the same time, the EDS analysis was also applied to CMC/CuO-TiO_2_ beads. Elements such as Cu, Ti, O, C, Cl, and Al were all detected in the CMC/CuO-TiO_2_ bead spectra (Figure 4b,b’), which indicated that the beads containe these elements. The production of CMC/CuO-TiO_2_ beads was confirmed by these peaks. Cu, Ti, O, and C were due to CuO-TiO_2_ and CMC, whereas Al and Cl were existing due to the cross-linking agent (AlCl_3_). EDS proved that the developed CMC/CuO-TiO_2_ beads were successfully prepared and contained Cu, Ti, O, C, Al, and Cl.

### 3.2. Catalytic Reduction

#### 3.2.1. Reduction of Nitrophenol Isomers

**Figure 5:**
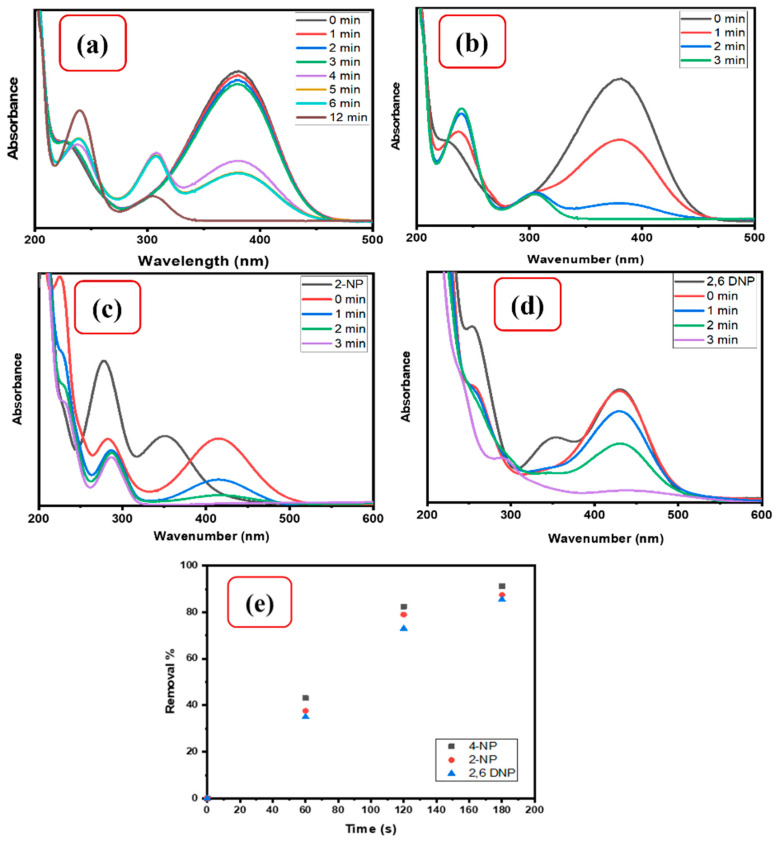
(**a**) UV–Vis spectra for 4-NP reduction using 5 mg CuO-TiO_2_ in the presence of NaBH_4_, (**b**–**d**), 4-NP, 2-NP, and 2,6-DNP reduction using 5 mg CMC/CuO-TiO_2_ in the presence of NaBH_4_, (**e**) removal percentage of 4-NP, 2-NP and 2,6-DNP using 5 mg CMC/CuO-TiO_2_ in the presence of NaBH_4_.

The CMC/CuO-TiO_2_ nanocatalyst beads were initially investigated for their ability to reduce 4-NP in the presence of NaBH_4_. As can be observed in Figure 5, the 4-NP has a pale yellow absorbance peak at 317 nm. The color changed to brilliant yellow after adding 0.5 mL of 0.1 M NaBH_4_, and the absorbance band was shifted to a longer wavelength of 400 nm at the same time. This is frequent in 4-NP reduction; a change in color and a shift in the absorbance band are indications of 4-nitrophenolate ion formation. However, in the absence of a catalyst, the additional conversion of 4-nitrophenolate ions to colorless amino phenol (4-AP) by NaBH_4_ takes a long time, and for a few dyes, even with a large amount of reducing agent, it is not accomplished [43]. A fast and good catalytic reduction of 4-NP with only NaBH_4_ would be good enough; unfortunately, it seems to be impossible to achieve it without a catalyst. Thus, a fast and good catalytic reduction of 4-NP with only NaBH_4_ (without a catalyst) cannot be carried out. The excellent reduction of 4-NP requires an efficient catalyst in the presence of NaBH_4_, which can speed up the reduction reaction. Therefore, CuO-TiO_2_ nanocatalyst was tested for the reduction of 4-NP to 4-AP. After the introduction of CuO-TiO_2_ to the mixture, the absorbance band intensity at 400 nm decreased, with the gradual disappearance of the bright yellow color. At the same time, a new absorbance band appeared at 300 nm. These results were signs of the complete reduction of the nitro group (-NO_2_) in 4-NP to an amine group (-NH_2_). Figure 5a shows the changes taking place during the catalytic reduction of 4-NP by CuO-TiO_2_, where the reduction occurs in 12.0 min according to the spectra. However, the novel nanocatalyst beads CMC/CuO-TiO_2_ were also evaluated for the catalytic reduction of 4-NP. As Figure 5a confirmed that CMC/CuO-TiO_2_ performed a good catalytic reduction of 4-NP to 4-AP in a shorter period of time compared to CuO-TiO_2_, where the catalytic reduction of 4-NP to 4-AP was accomplished in only 3.0 min. The fast reduction ability of CMC/CuO-TiO_2_ might be due to the contribution of the Al^+3^ ion in the reduction, which had been used as a crosslinker in the preparation of beads.

Similarly, under the same procedure described above, the catalytic behaviors of CMC/CuO-TiO_2_ nanocatalyst beads were also tested for the catalytic reactions of nitrophenol isomers such as 2-NP and 2,6-DNP into their corresponding amino groups in the presence of 0.5 mL of 0.1 M NaBH_4_. Figure 5 indicates that pure 2-NP and 2,6-DNP had strong absorbance bands at 317 nm and 428 nm, respectively. In the beginning, the catalytic reduction was examined in the absence of the nanocatalyst beads. The color of the nitrophenol isomers (2-NP and 2,6-DNP) changed from pale yellow to bright yellow, besides a slight shift of both absorption bands with only excess NaBH_4_. However, using 5 mg of CMC/CuO-TiO_2_ nanocatalyst beads, the 2-NP and 2,6-DNP were reduced to 2-AP and 2,6-DAP, respectively. The intensity of the absorbance peak at 413 and 428 nm steadily dropped as the process progressed, while a new absorbance band developed at 280 nm with increased intensity (Figure 5c,d). The spectra revealed that the reduction reaction of 2-NP and 2,6-DNP occurred within 3.0 min. The removal percentage of the reduction process was estimated using Equation (1). The reduction of nitrophenol isomers 4-NP, 2-NP, and 2,6-DNP were found to be 90.24%, 87.5%, and 85.43%, respectively. The CMC/CuO-TiO_2_ nanocatalyst was efficient, selective, and had excellent catalytic activity toward nitrophenol isomers.

**Figure 6:**
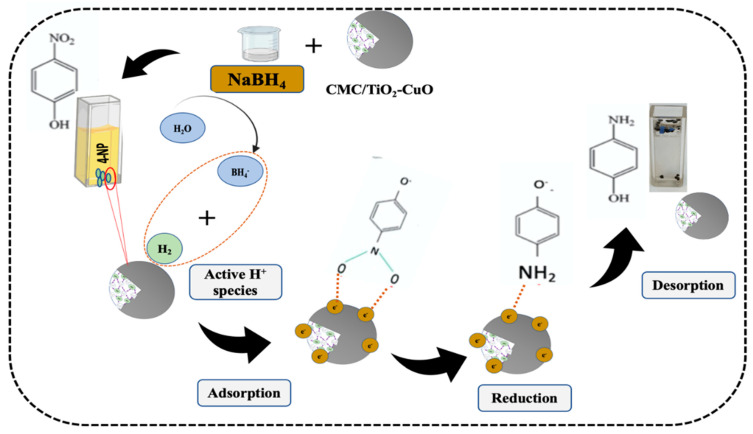
Schematic representation for the catalytic reduction of 4-NP.

Figure 6 depicts the schematic representation of 4-NP reduction mechanism. In accordance with this scheme, it is proposed that initially both BH_4_*^−^* and 4-NP get adsorbed on the surface of the catalyst. The catalyst enhances the transfer of electron from BH_4_*^−^* to 4-NP and thus decreases the activation energy of the reaction.

##### Effect of 4-Nitrophenol Concentration

**Figure 7:**
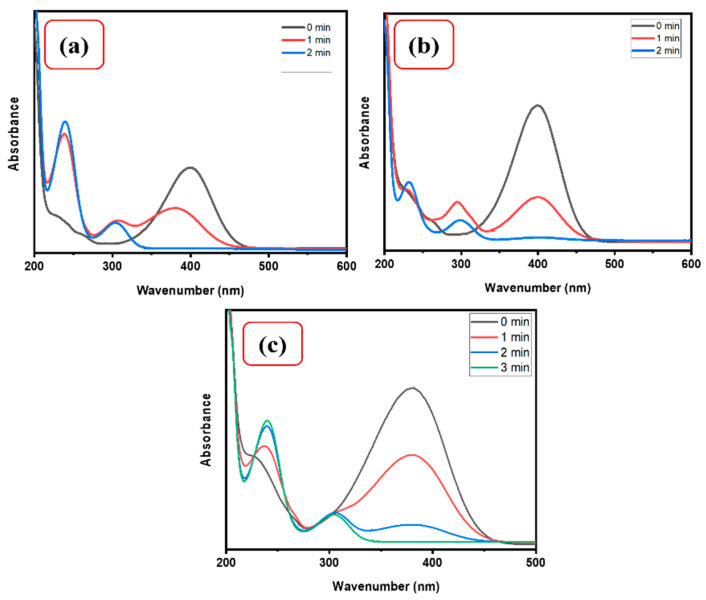
UV–Vis spectra for (**a**) 0.04, (**b**) 0.08, and (**c**) 0.13 mM 4-NP using 5 mg CMC/CuO-TiO_2_ in the presence of NaBH_4_.

The effect of 4-NP concentration on the catalytic activity of CMC/CuO-TiO_2_ was studied using 0.1 M NaBH_4_. For this investigation, three different concentrations of 4-NP solution (0.13, 0.08, and 0.04 mM) were prepared to determine the catalytic effects of 4-NP concentration. Figure 7 indicated the spectra of a range of 4-NP concentrations, in which 0.13 mM concentration reduced up to 90% in 3.0 min and up to 83% and 80% in 2.0 and 1.30 min for 0.08 and 0.04 mM, respectively. As a result, it was noticed that as compared to higher concentrations of 4-NP, the low concentration could be easily reduced while the catalyst quantity remained constant [1].

##### Effect of NaBH_4_ Concentration

**Figure 8:**
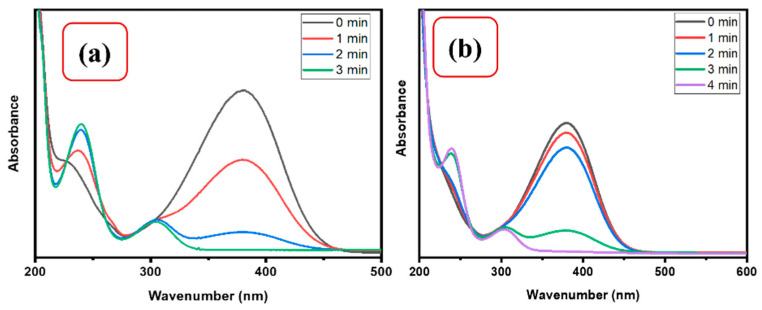
UV–Vis spectra for 0.13 mM 4-NP in the presence of (**a**) 0.5 M and (**b**) 0.1 M NaBH_4_ using 5 mg CMC/CuO-TiO_2_.

The influence of NaBH_4_ concentration on the catalytic reduction of 4-NP was studied by using CMC/CuO-TiO_2_ nanocatalyst beads. Various doses of NaBH_4_ (0.1 M and 0.05 M) were employed in the presence of 5 mg CMC/CuO-TiO_2_ beads in this work. Based on the results, increasing the NaBH_4_ concentration led to speeding up the reaction, as shown in the UV–Vis spectra of the catalytic reduction of 4-NP in Figure 8. With 0.1 M and 0.05 M, 4-NP was reduced up to 90% and 92%, respectively. As a result, it was shown that 0.1 M NaBH_4_ concentration could speed up the reaction and reduce the 4-NP in less time than 0.05 M concentration of NaBH_4_.

#### 3.2.2. Reduction of Organic Dyes

**Figure 9:**
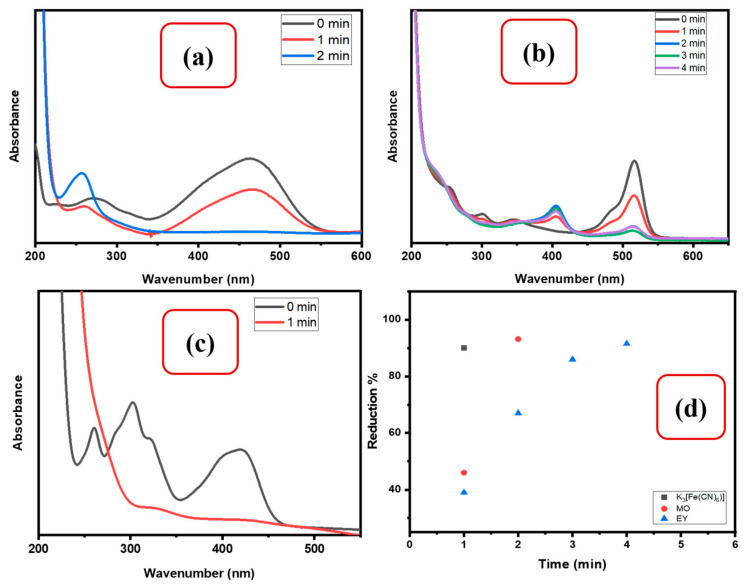
UV–Vis spectra for catalytic reduction of (**a**) MO, (**b**) EY, and (**c**) K_3_[Fe(CN)_6_] using CMC/CuO-TiO_2_ beads in the presence of NaBH_4_, (**d**) removal percentage of MO, EY, and K_3_[Fe(CN)_6_] using 5 mg CMC/CuO-TiO_2_ in the presence of NaBH_4_.

Organic dyes are widely used in the textile industry, and their non-biodegradability, toxicity, mutagenicity, and carcinogenicity make them a growing source of pollution in the environment. The goal of this investigation was to see how well the newly designed nanocatalyst beads reduce two types of organic dyes: methyl orange (MO) and eosin yellow (EY). UV–Vis spectroscopy was used to record the catalytic reduction, as shown in Figure 9a,b.

The catalytic reduction of MO and EY was tested using 5 mg of CMC/CuO-TiO_2_ nanocatalyst beads in the presence of NaBH_4_. In the addition of only NaBH_4_, no reductive reaction could take place between NaBH_4_ and the dyes. However, a regular decrease in the adsorption band was observed in the presence of CMC/CuO-TiO_2_ nanocatalyst beads. The catalytic reduction was recorded via UV–Vis spectroscopy. As clearly seen from (Figure 9a,b), the removal (%) of EY and MO occurred within 1.0–4.0 min, which reduced MO and EY up to 93.14%, and 91.5%, respectively (Figure 9d). Electrons were transferred from BH_4_*^−^* to CMC/CuO-TiO_2_ and then to acceptor dye molecules, reducing the azo (-N=N-) group found in dye molecules, according to recent results [1].

#### 3.2.3. Reduction of Inorganic Complex

Inorganic contaminants such as K_3_[Fe(CN)_6_] are known as pollutants that are distributed in the environment in either soil or water. Accordingly, K_3_[Fe(CN)_6_] can cause acute toxicity and carcinogenicity at very low levels, and it has been proven to easily accumulate inside human beings via food chains [44,45]. Due to this, K_3_[Fe(CN)_6_] was chosen as one of our selected pollutants. To test the catalytic activity of CMC/CuO-TiO_2_ beads, they were used to catalyze the reduction of K_3_[Fe(CN)_6_]. The UV–Vis absorbance of the catalytic reduction of K_3_[Fe(CN)_6_] was monitored to check the progress of the K_3_[Fe(CN)_6_] reaction. Using CMC/CuO-TiO_2_, the absorption band of K_3_[Fe(CN)_6_] at 420 nm was gradually lowered in 1.0 min, with the yellow color disappearing, indicating the complete reduction of K_3_[Fe(CN)_6_] to K_4_[Fe(CN)_6_] [44]. Figure 9c shows the transformation of 90% of K_3_[Fe(CN)_6_] to K_4_[Fe(CN)_6_], which was obtained in only 1.0 min.

An electron-transfer reaction is the probable mechanism for the reaction of K_3_[Fe(CN)_6_] in the presence of catalyst beads and NaBH_4_, as indicated in Equation (2) below [1,44].

BH_4_^−^ (aq)+ 8[Fe(CN)_6_]^−3^ (aq) + 3H_2_O (aq) → H_2_BO_3_^−^ (aq) + 8[Fe(CN)_6_]^−4^ (aq) + 8H^+^ (2)

As a result, there are two steps in the catalytic reaction mechanism of K_3_[Fe(CN)_6_]. The reducing agent NaBH_4_ causes the polarization of the catalyst nanocomposite beads at first. Subsequently, electrons are transported from the catalyst surface to the [Fe(CN)_6_]^−3^ pollutant, where they are reduced to [Fe(CN)_6_]^−4^. The obtained reduction results of all studied compounds were compared with the literature, as shown in Table 1 [1,6,18,27,42,46,47,48].

#### 3.2.4. Catalyst Stability and Reusability

**Figure 10:**
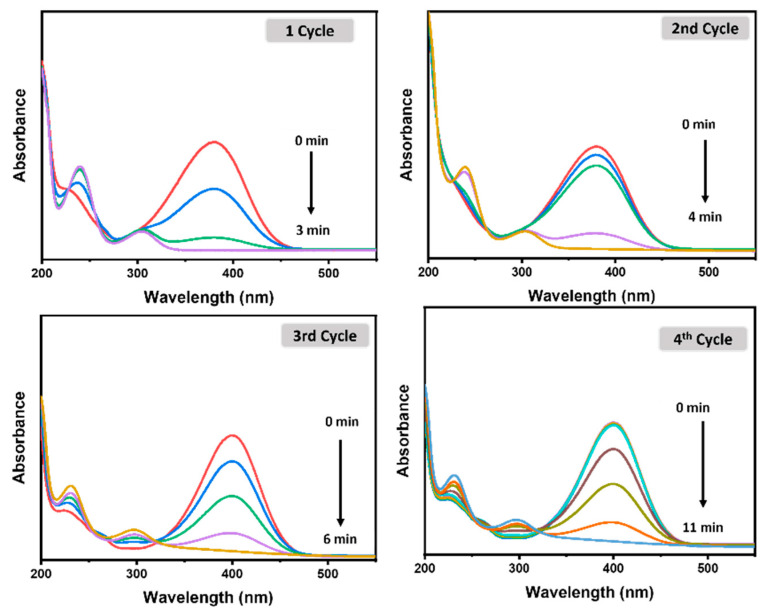
UV–Vis spectra for recyclability of CMC/CuO-TiO_2_ beads toward the catalytic reduction of 0.13 mM 4-NP.

Besides the activity of catalysts, stability is also a critical factor for evaluating their efficiency and potential applications. For this study, the stability of CMC/CuO-TiO_2_ nanocatalyst beads was examined regarding the catalytic reduction of 4-NP, and we found that the beads were stable for up to more than one year without any degradation or loss of activity. The recyclability of CMC/CuO-TiO_2_ beads was also studied for the catalytic reduction of 4-NP under the same conditions described in the experimental part. The CMC/CuO-TiO_2_ nanocatalyst beads were examined over four cycles, and the beads were washed three times after each use with distilled water and dried at RT. The catalytic reduction of 4-NP took 3.0 min for the first use, and 4.0 min, 6.0 min, and 11.0 min for the second, third, and fourth cycles, respectively (Figure 10). This suggests that the catalyst was active and effective in the reduction of 4-NP. Thus, the CMC/CuO-TiO_2_ beads were able to be used up to four cycles.

#### 3.2.5. Catalytic Efficiency of CMC/CuO-TiO_2_ Beads in Real Samples

To evaluate the efficiency of the CMC/CuO-TiO_2_ nanocatalyst beads under optimized conditions, four types of real samples were used (orange juice, full-fat milk, seawater, and wastewater), which were collected or obtained from a local market (Jeddah, Saudi Arabia). Real samples were prepared by diluting approximately 1 mL of each sample in 100 mL of deionized water individually. Afterwards, 2.5 mL of each sample was then transferred into a cuvette cell, together with 0.5 mL of 0.13 mM 4-NP, 0.5 mL of 0.1 M NaBH_4_, and 5 mg of CMC/CuO-TiO_2_ nanocatalyst beads. A UV–Vis spectrophotometer was used to monitor the catalytic reduction of 4-NP. As can be observed from the data in Table 2, full-fat milk and seawater have low reduction percentages, which reduced by up to 79.4% in 10.0 min and 82.5% in 11.0 min, respectively. This is because there was a lot of interference in these samples, which can impact the catalytic reduction of 4-NP. The reduction of 4-NP in the other samples took 5.0 min and was 92–89%. The results showed that the CMC/CuO-TiO_2_ nanocatalyst was effective in decolorizing and reducing of 4-NP from the real samples.

## 4. Conclusions

Herein, a simple and potentially cost-effective method was used for the fabrication of nanocatalyst CMC/CuO-TiO_2_ beads. The CMC/CuO-TiO_2_ beads were analyzed using SEM, XRD and EDS. The developed beads exhibited high catalytic activity toward nitrophenol isomers (4-NP, 2-NP, and 2,6-DNP), organic dyes (MO and EY), and inorganic complex K_3_[Fe(CN_6_)]. The developed materials can act as promising nanocatalysts for water treatment purposes.

## Figures and Tables

**Table 1:** Comparison of the catalytic reduction of 4-NP, MO, EY, and K_3_[Fe(CN)_6_] by using CMC/CuO-TiO_2_ beads in the presence of NaBH_4_ with other reported catalysts.

Pollutant	Catalyst	Time (s)	Reference
4-NP	CMC/CuO-TiO_2_	180	This Work
4-NP	Co-Cu/CIN/SCMC/TiO_2_	240	[27]
4-NP	*cf*-CA-AuNPs	900	[46]
4-NP	Fe_3_O_4_/TiO_2_/CuO	120	[18]
MO	CMC/CuO-TiO_2_	120	This Work
MO	CA-ZA10@Ni NPs	1080	[47]
MO	Ni/Cs@CMC/CuO-Co_2_O_3_	120	[42]
EY	CMC/CuO-TiO_2_	240	This Work
EY	Ni/Cs@CMC/CuO-Co_2_O_3_	360	[42]
EY	MnFe_2_O_4_@PANI@Ag	420	[48]
K_3_[Fe(CN)_6_]	CMC/CuO-TiO_2_	60	This Work
K_3_[Fe(CN)_6_]	Ni/Cs@CMC/CuO-Co_2_O_3_	360	[42]
K_3_[Fe(CN)_6_]	Alg@Cu_2_O-Sb_2_O_3_	180	[6]
K_3_[Fe(CN)_6_]	CMC/CuO-NiO	40	[1]

**Table 2:** Application of CMC/CuO-TiO_2_ nanocatalyst beads on four types of real samples spiked with 4-NP.

Real Sample	Reduction Time (min)	% Reduction
Orange Juice	5.0	92.6%
Full-Fat Milk	10.0	79.4%
Wastewater	5.0	89.2
Seawater	11.0	82.5%

## Data Availability

The data presented in this study are available on request from the corresponding author.
